# Effectiveness of HPV vaccination against the development of high-grade cervical lesions in young Japanese women

**DOI:** 10.1186/s12879-020-05513-6

**Published:** 2020-11-05

**Authors:** Yuki Shiko, Ryo Konno, Hiroshi Konishi, Catherine Sauvaget, Yasuo Ohashi, Tadao Kakizoe

**Affiliations:** 1 Biostatistics Section, Clinical Research Center, Chiba University Hospital, 1-8-1 Inohana, Chuo-ku, Chiba, 260-8677 Japan; 2 Department of Obstetrics and Gynecology, Jichi Medical University Saitama Medical Center, Omiya-ku, Saitama, 330-8530 Japan; 3 Japan Cancer Society, Tokyo, Japan; 4 Screening Group, Early Detection and Prevention Section, International Agency for Research on Cancer (WHO), Lyon, France; 5 Department of Integrated Science and Engineering for Sustainable Society, Chuo University, Tokyo, Japan

**Keywords:** Human papillomavirus, Vaccine effectiveness, Cervical intraepithelial neoplasia, Organised screening programme, Japan

## Abstract

**Background:**

Although more than 10 years have passed since HPV vaccination was implemented, first as an interim programme (Emergent vaccine promotion programme) in November 2010, followed by incorporating into the National Immunization Programme in April, 2013 and suspended in June 2013, limited studies have investigated the HPV vaccine effectiveness against high-grade cervical lesions in Japan.

**Methods:**

We collected the matched data of the results of cervical biopsy and history of vaccination from the Japan Cancer Society database. The subjects were women aged 20 to 29 years screened for cervical cancer between April, 2015 and March, 2017, and with information on HPV vaccination status. We estimated the relative risk of developing high-grade cervical lesions in vaccinated subjects using Poisson regression as compared to unvaccinated subjects.

**Results:**

Among the 34,281 women screened, 3770 (11.0%) were vaccinated. The prevalence of CIN2+ was statistically significantly lower in the vaccinated women as compared to the unvaccinated women (Vaccine Effectiveness (VE) =76%; RR = 0.24, 95% CI:0.10–0.60). High VE against CIN3+ was also observed (91%; RR = 0.09, 95% CI:0.00–0.42).

**Conclusion:**

Women aged 20–29 years who received at least one dose of HPV vaccine had a significantly lower risk of high-grade cervical lesions than those not vaccinated. In Japan, HPV vaccination should be resumed in order to reduce the incidence of cervical cancer.

## Background

The incidence rate of cervical cancer in developed countries is generally lower than that in resource-constrained settings. However, the estimated age-standardized incidence rate of cervical cancer in Japan is higher than other developed countries (14.7 per 10^5^ person-years in Japan; 6.5 in USA; 8.4 in UK) [1], but it is comparable to that in low- and middle-income countries such as India (14.7) or in The Philippines (14.9) [1]. This incidence rate is partly explained by the lower participation to cervical cancer screening (overall: 35.8%, age 20–24: 15.1%, 25–29: 36.6%) as compared to that of developed countries (based on the Comprehensive Survey of Living Conditions, 2019 [2]). Moreover, the age-standardized mortality rate is also higher than the rates in countries of similar economical level (2.7 per 10^5^ person-years in Japan; 1.9 in USA; 1.7 in UK) [1].

Persistent infection with human papillomavirus (HPV) can cause cervical lesions and cervical cancer [3, 4], while high efficacy of HPV vaccine has been demonstrated in clinical trials [5–8]. Bivalent, quadrivalent and nonavalent HPV vaccines are incorporated into National Immunization Programmes (NIPs) and they had already been introduced in 96 countries by June 2019 [9]. The nonavalent vaccine that can prevent infection by 90% of the oncogenic HPV strains has been approved in July, 2020 in Japan [6].

The bivalent vaccine, which showed 93.2% vaccine efficacy against CIN3+ in global clinical trial [10], was licensed in Japan in October 2009, and an interim national programme (Emergent vaccine promotion programme) started in November 2010, followed by inclusion in the NIP and given for free to girls aged 12–16 years old from April 2013. However, after numerous media reports on adverse events following HPV vaccination [11], the Japanese Ministry of Health, Labor and Welfare (MHLW) suspended proactive recommendation for the vaccine in June 2013 as a precautionary measure [12]. Although a recent epidemiological study from a Japanese team reported that there was no causal association between the vaccine and reported symptoms or adverse events [13], MHLW is persisting in suspending the HPV vaccine proactive recommendation. In settings where the screening programme shows adequate performance indicators, incidence and death rates are not likely to rise even if HPV vaccination is not implemented. However, the Japanese current combined situation of suspension of HPV vaccine proactive recommendation and low screening uptake is likely to fail to reduce the cervical cancer burden [14].

Numerous clinical trials [5–8] and epidemiological studies [15–21] showed strong efficacy and effectiveness of HPV vaccine. In Japan, 10 years have passed since HPV vaccine started as an interim national programme, adolescent girls who have received the public HPV vaccine have attained the age of 20 years or older; 20 years old being the starting target age of the cervical cancer screening programme. Several studies reported the vaccine effectiveness against cervical cytological abnormalities [22, 23]. Our earlier study investigated 22,743 women who were screened for cervical cancer between April, 2015 and March, 2016. The vaccine effectiveness against histologically confirmed high-grade cervical lesions, CIN 2 or worse (CIN2+) was 69% (RR = 0.31, 95% CI: 0.08–0.80), but effectiveness against CIN3+ could not be estimated due to the limited sample size [24]. Because of the lack of national vaccine registry and national screening registry in Japan, we cannot use the national database in order to evaluate the vaccine effectiveness in the “real world”. In place of the national database, we took advantage of the JCS database, the largest database on CIN and screening of the country. This update was therefore conducted to increase the CIN3+ sample size. The purpose of this study was to investigate the effectiveness of HPV vaccine against histologically confirmed CIN2+ and CIN3+ in young women aged 20 to 29 years who underwent cervical cancer screening between April, 2015 and March, 2017.

## Methods

### Study design and data sources

We conducted a cross-sectional study which is an update of our earlier study, to investigate the effectiveness of HPV vaccine against histologically confirmed high-grade cervical lesions at the screening visit (*n* = 37,505). The Japan Cancer Society (JCS) with 46 branches (equivalent of representative offices) among 47 prefectures nationwide, is the Japan’s largest cancer screening organization, screening more than 11 million people every year. Some branches collect information on vaccination history, results of screening (cytology and if biopsy done, pathological results) and grade of cervical lesion. Pathological diagnosis is reported according to the WHO 2014 classification [25] and CIN classification. Women fill a self-reported questionnaire with information on vaccination history at the time of cervical cancer screening. In the present study, we collected the data from 26 branches of JCS. Among 26 branches, 7 branches did not inquire about vaccination history at screening and they were excluded. So, we finally included the data of 19 branches. The study subjects were women aged 20 to 29 years who underwent cervical cancer screening in the FY 2015 (April 2015 to March 2016) and FY2016 (April 2016 to March 2017). We defined women who received at least one dose of HPV vaccine as vaccinated. The study outcomes are histologically confirmed diagnosis of CIN2+ and CIN3 + .

### Statistical analyses

The analysis was performed in two parts. In the first analysis, to evaluate the effectiveness of HPV vaccine against CIN2+ and CIN3+ for women aged 20–29 and 20–22, we estimated the relative risk (RR) and associated 95% confidence interval (CI) for vaccinated subjects using Poisson regression as compared to the unvaccinated subjects. We adjusted for age as fixed effect and place of screening (i.e. JCS branch) as random effect. For CIN3+, the number of events was small, RR and associated 95% CIs were estimated using exact Poisson regression for aged 20–29. We adjusted for age as fixed effect in this model. Vaccine effectiveness (VE) was calculated as: (1- adjusted RR) × 100.

In the MHLW guidelines, the recommended screening interval of cervical cancer is 2 years; actually, however, the intervals of screening vary depending on the local governments responsible of the cancer screening organization. Therefore, some women might have undergone cervical cancer screening in both FY2015 and FY2016, i.e. within less than the 2-year interval of the recommended national guidelines. We defined this fact as “overlapping”. Therefore, the main analysis was performed after removing this overlapping in FY2016 (3024 women (15.6%) screened in both FY2015 and FY2016). Statistical analyses were performed using the SAS statistical software package, Version 9.4 (SAS Institute, Cary, NC, USA).

## Results

The characteristics of the subjects are shown in Table 1. Among the 37,305 women aged 20–29 years, 4083 (10.9%) were vaccinated. The vaccination rates of 20, 21 and 22 years that correspond to an interim national vaccination recipient were high at 62.7, 44.6 and 23.8%, respectively, and 2.6 to 7.9% after 24 years. In the vaccinated subjects, the total number of cases of CIN2+ was only 7 (0.17%) with no CIN3+. In the unvaccinated subjects, the cases of CIN2+ and CIN3+ were 188 (0.57%) and 78 (0.23%), respectively.

**Table 1:** Information on vaccination status, CIN2+ and CIN3+, among women aged 20–29 years (with overlapping)

Age at screening (year)	Vaccine(−)	Vaccine(+)		CIN2+		CIN3+	
				Vaccine(−)	Vaccine(+)	Vaccine(−)	Vaccine(+)
	n	n	%	n	n	n	n
20	522	878	62.7	4	1	1	0
21	1860	1496	44.6	6	1	3	0
22	1569	489	23.8	8	1	2	0
23	2517	217	7.9	17	1	8	0
24	2985	214	6.7	11	1	4	0
25	3035	149	4.7	17	0	6	0
26	5367	189	3.4	34	1	14	0
27	3849	128	3.2	20	0	7	0
28	6820	184	2.6	50	0	22	0
29	4698	139	2.9	21	1	11	0
Total	33,222	4083	10.9	188	7	78	0

After removal of overlapping, we included 34,281 women in the analyses (Table 2). In the vaccinated subjects, the cases of CIN2+ were 5 (0.13%) and no CIN3+. In the unvaccinated subjects, the cases of CIN2+ and CIN3+ were 182 (0.59%) and 77 (0.25%), respectively.

**Table 2:** Vaccination status, CIN2+ andCIN3+, among women aged 20–29 years (without overlapping)

Age at screening (year)	Vaccine(−)	Vaccine(+)		CIN2+		CIN3+	
				Vaccine(−)	Vaccine(+)	Vaccine(−)	Vaccine(+)
	n	n	%	n	n	n	n
20	514	869	62.8	4	1	1	0
21	1822	1436	44.1	5	1	3	0
22	1435	399	21.8	8	0	2	0
23	2367	197	7.7	17	1	8	0
24	2710	189	6.5	11	1	4	0
25	2740	115	4.0	17	0	6	0
26	4998	175	3.4	33	1	14	0
27	3428	108	3.1	20	0	7	0
28	6354	165	2.5	48	0	22	0
29	4143	117	2.8	19	0	10	0
Total	30,511	3770	11.0	182	5	77	0

The relative risk of developing high-grade cervical lesions according to the vaccination status is shown in Table 3. Vaccine effectiveness against CIN2+ and CIN3+ was 67% (RR = 0.33, 95% CI = 0.15–0.73) and 91% (RR = 0.09, 95% CI = 0.00–0.41), respectively. High vaccine effectiveness against CIN2+ was also observed for those aged 20–22 years old (VE = 77%; RR = 0.23, 95% CI = 0.06–0.82).

**Table 3:** Relative risk of developing CIN2+ and CIN3+ lesions among vaccinated women as compared to the unvaccinated women

	CIN2+			CIN3+		
	RR(95%CI)		*P*-value	RR(95%CI)		*P*-value
Aged 20–29						
Unvaccinated	1.00	Reference		1.00	Reference	
Vaccinated	0.33	(0.15, 0.73)	0.006	0.09	(0.00, 0.41)	0.002
Age	1.04	(0.98, 1.10)	0.233	1.06	(0.00, 1.17)	0.246
Aged 20–29 (no overlapping)						
Unvaccinated	1.00	Reference		1.00	Reference	
Vaccinated	0.24	(0.10, 0.60)	0.003	0.09	(0.00, 0.42)	0.002
Age	1.03	(0.97, 1.09)	0.360	1.06	(0.96, 1.17)	0.246
Aged 20–22						
Unvaccinated	1.00	Reference				
Vaccinated	0.23	(0.06, 0.81)	0.023			
Age	0.95	(0.51, 1.76)	0.869			
Aged 20–22(no overlapping)						
Unvaccinated	1.00	Reference				
Vaccinated	0.16	(0.03, 0.72)	0.017			
Age	0.89	(0.47, 1.68)	0.709			

In the main analysis (without overlapping), the vaccine effectiveness against CIN2+ and CIN3+ in the age-group 20–29 was 76% (RR = 0.24, 95% CI = 0.10–0.60) and 91% (RR = 0.09, 95% CI = 0.00–0.42), respectively. Vaccine effectiveness against CIN2+ was also observed for those aged 20–22 years old (VE = 84%; RR = 0.16, 95% CI = 0.03–0.72). The age effect was not significant in all analyses.

## Discussion

In the present study, we compared the prevalence of histology confirmed CIN2+ and CIN3+ between an HPV-vaccinated subjects and unvaccinated subjects. As a result, the prevalence of CIN2+ and CIN3+ was significantly lower in the vaccinated subjects as compared to the unvaccinated subjects.

Population-based studies are also conducted in many countries [15–21]. In Scotland, the risk of CIN3+ following bivalent vaccine at age 20 years was reduced by 86% in women who were vaccinated at the age of 12–13 years old [21]. In Sweden, the risk of CIN2+ and CIN3+ following quadrivalent vaccine was reduced by 75 and 84% in women who were vaccinated before the age of 16 years old [16]. These results were based on vaccination in women with 3 doses [16, 19], while our results were based on vaccination with at least one dose, because we could not obtain information on the number of doses. Several studies focused on the effectiveness of the number of doses received on CIN occurrence [26, 27]. In a case-control study from Australia, vaccine effectiveness for CIN2+ was observed in both 2- and 3-dose recipients (VE = 46% in the 3-dose recipients, VE = 21% in the 2-dose recipients) [27]. In the database linkage study from Australia, the vaccine effectiveness against high-grade was observed in the 3-dose recipients (hazard ratio = 0.86, 95% CI: 0.78–0.94) and, women who were vaccinated before the age of 16 years old, trends of effectiveness were observed in less than 3-dose recipients [26]. A recent publication from India reported a rate of CIN1+ of 4.5% (5/132) in unvaccinated subjects, while there were no case (0/24) in vaccinated women (2- and 3-dose) [28]. These studies confirm that less than 3-dose regimens of HPV vaccine are effective against CIN, leading to the current WHO recommendation on administration of a 2-dose schedule, in subjects aged less than 13 or 14 years old depending on the vaccine (9–13 years old with the quadrivalent vaccine, 9–14 years old with the bivalent and nonavalent vaccines) [29].

Moreover, in recent reports from Denmark and Australia (with high coverage), one-dose regimen showed similar effectiveness than 3-dose regimen [30, 31]. Considering the results of these studies, it might be reasonable to support that high effectiveness of vaccine was observed in the present study.

Several limitations should be acknowledged. First, information on important confounding factors such as sexual behavior and healthcare seeking behavior were not available; consequently, the effect of these potential confounding factors could not be controlled. In addition, information on HPV types and specificity and sensitivity of CIN2+ and CIN3+ from each branch of JCS were not available. Second, HPV vaccination status was self-reported and might be affected by recall bias. Finally, the biggest limitation is that Japan has neither national vaccine registry nor national screening registry. Therefore, it is difficult to collect history of vaccination and screening results of individuals, and to link these data is even more difficult [32]. Deployment of the epidemiological surveillance at the national level is one of the most important challenges in the public health policy in Japan. Previous other studies in Japan were based on limited sample size, and on cytology results solely [22, 23]. Our early study using the data of 22,743 women from JCS showed the statistical effectiveness of the vaccine against CIN2 + only [24]. This time, we can report the statistically high effectiveness of vaccine against both CIN2+ and CIN3+, because we collected the linked data of 37,505 women. This is the largest study ever conducted in Japan to our knowledge.

In Japan, MHLW suspended proactive recommendation for the vaccine, as a result, uptake rate for HPV vaccine was plummeted from 70% to 0.01% [12]. Although evidence of effectiveness and safety of vaccine has been accumulated, the recommendations have not yet been resumed. Additionally, screening uptake for cervical cancer in women younger than 30 is considerably low (2019, age 20–24 15.1%, 25–29 36.6%) [2]. Therefore, incidence rate is increasing especially among Japanese women aged 15–39 years old (1.69% per years between 1975 to 1994, 4.67% per year between 1994 to 2015) [33].

In Ireland, uptake rate for HPV vaccine has also declined due to concerns about vaccine safety, however the uptake rate has recovered owing to efforts such as social media and governmental campaigns [34]. Since cervical cancer is a preventable disease [35], it is important to take steps to improve the HPV vaccination uptake rate in order to discontinue the increase in incidence and mortality from cervical cancer.

The lessons learned from Japan are, unfortunately, that there is no linkage with other databases related to a screening programme such as the regional cancer registry, laboratory files, treatment files and vaccination registry. We strongly recommend an increase information technology system development to allow data collection and linkage of health data [32].

## Conclusions

We showed the high vaccine effectiveness against CIN2+ and CIN3+ in Japanese young women. We hope that HPV vaccination should be recommended actively as sooner as possible, therefore incidence and mortality rates from cervical cancer will stop increasing and will eventually decline.

## Data Availability

The datasets generated during and/or analyzed during the current study are available from the corresponding author on reasonable request.

## Abbreviations

CIN: Cervical Intraepithelial Neoplasia
HPV: Human papillomavirus
HSIL: High-grade squamous intraepithelial lesion
JCS: The Japan Cancer Society
MHLW: Ministry of Health, Labor and Welfare
NIP: National Immunization Programme
VE: Vaccine Effectiveness
